# Production of Vitamin D3-Fortified Plant-Based Meat Analogs Through High-Moisture Extrusion

**DOI:** 10.3390/foods14091500

**Published:** 2025-04-25

**Authors:** Lorena S. Pinho, Ramon P. Brexó, Tatielly de J. Costa, Marcelo Thomazini, Osvaldo H. Campanella, Carmen S. Favaro-Trindade

**Affiliations:** 1 College of Food, Agricultural, and Environmental Sciences, The Ohio State University (OSU), Columbus, OH 43210, USA; spinholorena@gmail.com (L.S.P.); campanella.20@osu.edu (O.H.C.); 2 The Good Food Institute (GFI), Washington, DC 20090, USA; 3 Faculdade de Zootecnia e Engenharia de Alimentos (FZEA), Universidade de São Paulo (USP), Pirassununga 13635-900, SP, Brazil; ramonperesbrexo@alumni.usp.br (R.P.B.); tatielly.costa@usp.br (T.d.J.C.); mthomazini@usp.br (M.T.)

**Keywords:** extrusion cooking, biosorption, cholecalciferol, brewer’s spent yeast, chemical stability, food fortification

## Abstract

Incorporating vitamin D3 (cholecalciferol) into food is hampered by its high instability and low water solubility. Due to porous structure that favors absorption and carrying of micronutrients, brewer’s spent yeast (BSY) is an economically and technically attractive alternative to overcome the shortcomings of vitamin D3 incorporation. Using heat and shear-sensitive ingredients and additives in formulations remains challenging due to the high-temperature and shear conditions during industrial processes, such as extrusion. This study aimed to produce an extruded plant-based meat product enriched with cholecalciferol. Vitamin D3, free and impregnated in BSY (BSY-VitD3), was blended with pea protein and subjected to cooking extrusion. Product features were analyzed for color, texture, moisture, water activity, absorption capacity, and vitamin retention. Adding BSY-VitD3 reduced all texture profile parameters and altered colors. Furthermore, free VitD3 enhanced extruded water and oil absorption capacity. After extrusion, vitamin retention percentages in the products were 45.4 and 91.6%, for free and BSY-VitD3, respectively. After 1-month storage of the extruded products, vitamin retention was 38.9 and 85.1% for free and BSY-VitD3 samples, respectively. Blending vitamin D3 with BSY is a simple, fast, and effective process to facilitate incorporation of the vitamin in the formulation and protect it during cooking extrusion.

## 1. Introduction

Vitamin D (VitD) exists in two primary forms: vitamin D2 (ergocalciferol) and vitamin D3 (cholecalciferol). Ergocalciferol is synthesized exclusively by plants, whereas cholecalciferol is produced by the human body, primarily through skin exposure to sunlight (UVB radiation) [1].

Despite the seemingly abundant sources of vitamin D, its deficiency remains one of the most common nutrient deficiencies globally. Several factors contribute to this public health issue, including geographic location (altitude and latitude), the sun’s angle and exposure duration, and environmental pollution [2,3]. Additionally, reduced sun exposure due to concerns about skin cancer and the limited availability of foods naturally rich in VitD exacerbate this deficiency. Major dietary sources of VitD include only a few wild varieties of mushrooms, certain types of algae, eggs, cod liver oil, salmon (*Salmo salar*), and other fatty fish like herring (*Clupea harengus*) and Atlantic mackerel (*Scomber scombrus*) [4,5].

To address the Recommended Dietary Allowance (RDA) of VitD—400–800 IU per day—many countries have authorized food fortification with VitD [6]. In the United States, cow’s milk is typically enriched with around 120 IU of vitamin D per cup, whereas in Canada, the required fortification level ranges from 35 to 40 IU. Similarly, plant-based milk alternatives—such as soy, almond, and oat beverages—are frequently fortified to match the vitamin D levels found in fortified dairy milk (approximately 3 µg [120 IU] per cup). Margarine, in particular, is mandated to contain a minimum of 530 IU. Additionally, vitamin D is commonly added to a variety of processed foods, including ready-to-eat breakfast cereals, selected brands of orange juice, yogurt, margarine, and other commercially available products [6]. However, fortifying foods with VitD presents challenges, such as low homogeneity in the food matrix, poor water solubility, vitamin degradation during processing and storage, and interactions with other food components that may alter sensory properties, impacting consumer acceptance and marketability [7]. One strategy to overcome these issues involves encapsulating VitD3 [3].

Various encapsulation techniques, including liposomes, nanostructured lipid carriers, emulsions, and spray drying, have been utilized to create efficient structures with desired functionalities, making them suitable for nutrient fortification like VitD [3]. Methods such as spray-chilling [8] and yeast biosorption [9] have also been explored. Our research group has successfully encapsulated VitD3 within brewer’s spent yeast (BSY), using a passive biosorption process followed by spray-drying [10], although this method proved to be time-consuming and somewhat energy deficient due to the significant energy used to evaporate water. Therefore, investigating faster and more sustainable alternatives for this process is of great importance.

Brewer’s spent yeast (BSY) is a byproduct of the brewing industry, with an average yield of 1.7 to 2.3 kg of yeast biomass per m^3^ of beer. Yeast biomass is removed at the end of bulk fermentation; while a small fraction is reused for subsequent fermentation, the majority is discarded as a byproduct [11]. BSY is rich in nutrients, particularly proteins, vitamins, and minerals, and contains functional and biologically active compounds such as polyphenols, antioxidants, beta-glucans, and mannoproteins [12,13]. Currently, BSY is mainly utilized as an inexpensive protein source for animal feed [13]. However, our group has previously demonstrated its potential as a carrier for encapsulating extracts from pumpkin and jaboticaba byproducts and grape pomace using a spray-drying process [14,15].

The permeability of BSY’s cell walls and plasma membranes makes it a promising carrier for bioactive molecules. Vacuum impregnation in a vacuum chamber is an innovative and efficient method for BSY impregnation compared to passive biosorption, offering a faster and cost-effective approach. This technique has been successfully applied to impregnate probiotic bacteria [16].

A promising application of vitamin D-fortified yeast lies in the development of plant-based meat alternatives via high-moisture extrusion (HME), which relies on various functional ingredients to replicate the texture, appearance, flavor, and mouthfeel of conventional animal proteins [17,18]. The plant-based meat sector is experiencing rapid growth, driven by increasing consumer demand for sustainable, health-focused food alternatives. Forecasts suggest a market valuation of up to USD 85 billion by 2030, highlighting its potential for continued expansion and innovation [17]. Pea protein stands out among the key raw materials used in plant-based meat analogs due to its availability and functional properties. Global pea cultivation spans approximately 2.18 million hectares, producing 21.77 million tons annually. Peas are particularly valuable in agriculture because of their ability to fix atmospheric nitrogen, improve soil fertility, and reduce the need for chemical fertilizers, making them a sustainable choice for plant-based protein sources [19]. Despite its favorable environmental impact and functional properties, pea protein has a lower content of certain essential amino acids than animal proteins, which may limit its nutritional adequacy [19,20].

Pea protein isolates and concentrates are often used as key ingredients for HME processes due to their gelling and fiber-forming abilities during heating and extrusion, as well as their oil-binding and emulsifying properties [17]. To replicate the sensory attributes and nutritional profile of meat, a variety of additives are incorporated into plant-based formulations, including yeast extract (for taste enhancement) and vitamins (for fortification) [17].

The extrusion strategy of combining pea protein with other nutrient-rich ingredients, such as brewer’s spent yeast (BSY), offers a promising solution. Extrusion involves the application of heat, pressure, and mechanical shear, which induces the textural transformation of plant proteins, enhancing their meat-like properties. The functional attributes of pea protein, such as its gelling, emulsifying, and fiber-forming capabilities, make it an ideal candidate for extrusion and subsequent nutrient fortification. Twin-screw extrusion is commonly employed in these products, allowing for continuous processes such as mixing, heating, shearing, and shaping plant proteins at high moisture levels, leading to the formation of a meat-like texture that is essential for consumer acceptance of meat alternatives as a fibrous and striated network [18,21].

In this context, the present study aimed to evaluate the impact of incorporating free cholecalciferol and cholecalciferol impregnated in BSY into formulating a fortified plant-based product. The novelty of this approach lies in combining the use of brewer’s spent yeast (BSY), an abundant and underutilized byproduct, as a bio-based carrier for vitamin D3, with its application in the development of plant-based meat analogs produced by extrusion—a combination not previously explored.

## 2. Materials and Methods

### 2.1. Materials

The ingredients for plant-based meat production included pea protein isolate (85% protein) provided by Roquette Frères (Lestrem, France), cholecalciferol (VitD3—98%) purchased from Sigma-Aldrich (Burlington, MA, USA), and *Saccharomyces pastorianus* spent biomass vacuum-impregnated with cholecalciferol from Sigma-Aldrich (675 µg VitD3/g yeast biomass).

Brewery Hausen Bier (Araras, São Paulo, Brazil) kindly provided the biomass of *Saccharomyces pastorianus* after its utilization five times in beer production. Merck Co. (Darmstadt, Germany) sourced all reagents used for High-Performance Liquid Chromatography (HPLC) analysis.

### 2.2. Methodology

#### 2.2.1. Saccharomyces Pastorianus Spent Biomass Preparation, Vacuum Impregnation, and Dehydration

Washing and plasmolysis of the yeast biomass were conducted following the [10] methodology. The yeast biomass was washed five consecutive times in distilled water and recovered by sedimentation every 24 h. This procedure was employed to remove suspended solids coming from the brewing process. To ensure storage stability, the washed yeast biomass was dried using a spray dryer (bench model MSD 1.0, Labmaq do Brasil Ltda, Ribeirão Preto, São Paulo, Brazil), with a feed rate of 0.8 L/h, 1.2 mm diameter nozzle, 140 °C air inlet temperature, 0.39 MPa compressor pressure, 100 °C outlet temperature, 2.5 m/s drying airflow, and 40 L/min compressor flow.

The resulting yeast biomass powder was then suspended in a 10% (*w*/*w*) sodium chloride (NaCl) solution and maintained at 55 °C and 180 rpm for 48 h. The cells were recovered by centrifugation (6000× *g* rpm, 10 min). The NaCl and any cytoplasmic materials in the supernatant were removed through five washes with distilled water. Finally, the plasmolyzed cell biomass was spray-dried and stored at −20 °C in a Schott flask.

The interaction of yeast biomass with cholecalciferol (from Sigma-Aldrich, USA) was facilitated using vacuum-assisted impregnation. A total of 0.3 g of dried plasmolyzed yeast biomass was suspended in 9.2 mL of distilled water, and 0.8 mL of an ethanolic solution of VitD3 (1 mg/mL) was added. The suspension was shaken in a Heidolph Multi Reax at 1500 rpm for 2 min. Subsequently, the mixture was placed in 15 × 20 cm boilable vacuum bags (TecMaq, São Paulo, SP, Brazil. The vacuum process was performed using a TecMac TM 100 Vacuum Sealer, applying full vacuum power with a holding time of 40 s [16].

After the vacuum process, biomass (BSY-VitD3) was collected by centrifugation (centrifuge Eppendorf, Hamburg, Germany) at 6000× *g* rpm for 5 min at 25 °C and then stored at −20 °C for subsequent spray drying, as detailed previously.

#### 2.2.2. Extrusion of Plant-Based Meat Analog Fortified with Vitamin D

A blend of pea protein and BSY-VitD3 (2%, *w*/*w*) or pea protein and free VitD3 (0.5%, *w*/*w*) was prepared in a mixer. These proportions were determined based on preliminary studies, which indicated that such concentrations are necessary to ensure efficient extraction and accurate quantification of vitamin D3 using a validated HPLC method. The preparation of extruded meat analogs involved three treatments: incorporation of free VitD3, incorporation of impregnated VitD3, and a control treatment without VitD3. They were produced using a Twin-Screw Extruder EV 32 (Clextral, Firminy, France). The extrudates were processed with a feed rate of 7.3 kg/h (41% *w*/*w* of pea protein:BSY-VitD3 or protein:VitD3 in final formulation), and water feed rate of 10.5 kg/h (59% *w*/*w* of water in final formulation), screw speed of 450 rpm, a melting zone temperature of 135 °C, and the temperature of a two-zone cooling die of 85 °C. After production, the meat analogs were vacuum packed and stored at −20 °C until further analysis.

#### 2.2.3. Extrudates Samples Characterizations

##### Texture

Meat analogs were cut into square pieces and then subjected to a two-bite test using a TAxT2i texture analyzer (manufactured by Stable MicroSystem, Godalming, UK). The samples were compressed with a P/30 probe to 50% of their original thickness for the first bite. After the first bite, the probe was returned to its original position, and a second bite was taken, again compressing the sample to 50% of the first compressed thickness. Tests results included hardness, resilience, cohesiveness, springiness, gumminess, and chewiness.

The same texture analyzer equipment was used to investigate the cutting forces in the samples’ parallel (F∥) or perpendicular (F⊥) direction. The Fibrous Degree (FD) was then expressed as the ratio of F⊥ to F∥.

##### Instrumental Color Parameters

The color of the meat analogs was assessed using a Minolta Colorimeter (Model CR-400, Minolta Co., Ltd., Osaka, Japan) to determine L* (lightness), a* (redness), b* (yellowness), c* (chroma), and h (hue angle) values. Measurements were randomly taken at various points on the samples, with each measurement performed three times. The color difference (ΔE) was calculated by Equation (1) [22]. The samples were also photographed to capture their appearance, illustrating the impact of VitD3 in the formulation.

(1) $$\Delta E=\sqrt{(L\;sample-L\;control{)}^{2}+(a\;sample-a\;control{)}^{2}+(b\;sample-b\;control{)}^{2}}$$

##### Water Activity and Moisture

Meat analogs were sliced into small pieces, and their water activity was measured using a water activity meter (Aqualab Series 4 TE, Decagon Devices Inc., Washington, DC, USA). Moisture content was determined by drying a 1 g sample at 105 °C for 24 h using an oven.

##### Water Absorption Capacity (WAC) and Oil Absorption Capacity (OAC)

To measure WAC, 1 g of the sample was mixed with 10 mL of distilled water, stirred using a vortex mixer for 30 s, and then allowed to sit for 30 min. The meat analogs soaked were centrifuged at 13,751× *g* for 30 min, the supernatant was discarded, followed by draining the samples for 5 min. The weight of the sediment was then measured. The same method was applied using cooking oil to measure OAC. WAC and OAC were calculated by Equation (2). The average value was determined based on three repetitions of each experiment.

(2) $$WAC\;or\;WOC=\frac{\left(Wsoaked-Wdry\right)}{Wsoaked}\times 100$$

#### 2.2.4. Assessment of VitD3 Retention During Processing and Storage

VitD3 retention in extruded samples was examined after the extrusion process and after 30 days of storage.

##### VitD3 Extraction of the Extruded

For the extraction procedure, approximately 1 g of each sample was precisely weighed in triplicate using an analytical balance (Ohaus, model AR2140, USA) and placed in 15 mL centrifuge tubes. The samples were then homogenized with 5 mL of methanol (Biograde, Anápolis, Goiás, Brazil). The extraction involved shaking at 1800 rpm for 5 min (Heidolph, Multi Reax model, Germany), followed by a 5 min ultrasonic bath (Unique/USC 1400, Indaiatuba, São Paulo, Brazil) and centrifugation at 2377× *g* for 5 min (centrifuge Eppendorf/5430R, Hamburg, Germany). The resulting supernatant was diluted 20-fold and passed through a 13 mm × 0.45-micron nylon membrane filter into 2 mL amber vials for liquid chromatography analysis. The VitD3 concentration in the samples was subsequently determined by HPLC.

##### HPLC VitD3 Quantification

The analytical conditions were adapted from the methodology described by [23]. A liquid chromatography system (Shimadzu, Prominence/Kyoto, Japan) controlled by LC Solution software version 1.25 was used. The system was equipped with a diode array detector at 265 nm and a reverse-phase C18 column with a 5 μm particle diameter (150 × 4.6 mm—Shimadzu, Shim-Pack VP-ODS/Kyoto, Japan) maintained at 30 °C. Methanol and acetonitrile were used as the mobile phase in a 90:10 (*v*/*v*) ratio, with a constant flow rate of 0.80 mL/min, 30 μL injection of the extract, and a total run time of 10 min. Identification was performed by comparing the retention time (7.01 min.) with the cholecalciferol standard (CAS Number 67-97-0; C27H44O, MM: 383.63 g/mol; Sigma-Aldrich, USA) and with the characteristic ultraviolet spectrum obtained between 200 and 380 nm. Quantification was carried out by external standardization in the working range of 2 to 70 µg/mL (Figure S1—Supplementary Material). The results were expressed in milligrams of cholecalciferol per 100 g of sample.

### 2.3. Statistical Analysis

The data were statistically analyzed using the SAS statistical software (Statistic Analysis System), version 8.02, by ANOVA and the Tukey test, at a 5% significance level.

## 3. Results and Discussion

### 3.1. The Plant-Based Meat Analogs

This study selected extruded plant-based meat analogs as the model food system for fortification with free vitamin D3 (VitD3) and vitamin D3 incorporated in BSY (BSY-VitD3).

**Figure 1:**
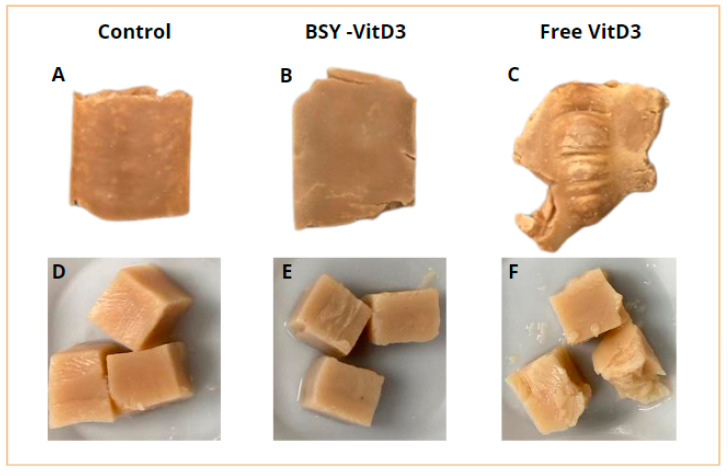
Appearance of Pea Extruded Samples. (**A**–**C**) illustrate the external appearance of the different extruded formulations, while (**D**–**F**) show their internal structure.

Plant-based meat products’ visual appearance and textural characteristics are critical for consumer satisfaction and market success [20,23]. As illustrated in Figure 1, the pea-based meat analogs fortified with 2% *w*/*w* vacuum-impregnated VitD3 (B) or 0.5% *w*/*w* free VitD3 (C) displayed a distinct appearance compared to the control sample (A) formulated with pea protein isolate only.

Samples including BSY-VitD3 exhibited a darker color and a more pronounced fibrous structure, both desirable attributes that closely mimic the appearance of traditional meat products. The vacuum impregnation technique is likely to enhance the distribution and retention of vitamin D3 within the BSY particles, contributing to improved stability during processing. On the other hand, passive biosorption using *Saccharomyces pastorianus* biomass as sorbent was also effective to reduce the degradation of vitamin C [24]. Although Costa et al. (2024) [10] employed a biosorption approach rather than vacuum impregnation, their results showed that structural modifications in plasmolyzed brewer’s spent yeast, such as increased porosity and intracellular space, favor the internal entrapment and retention of vitamin D3. These findings support the rationale that similar interactions may occur under vacuum impregnation, contributing to the stability observed in this study. Moreover, Zhao et al. (2023) [20] demonstrated that yeast-derived particles can be effectively integrated into plant-based protein matrices during high-moisture extrusion, which aligns with our results showing uniform incorporation and favorable vitamin retention in the extruded product.

Furthermore, the extruded formulation containing BSY-VitD3 exhibited a smoother and more homogeneous external surface (B) than the sample containing free VitD3 (C) and the control (A). Internally, the sample showed grooves indicative of increased stratification or fiber formation (E), although these features did not significantly differ from the control; both samples resembled the texture of raw chicken breast meat. In contrast, the formulation with free VitD3 displayed more pronounced changes, with an exterior surface (C) appearing wrinkled and irregular, and the interior (F) showing a brittle texture similar to that of cooked fish meat. This altered appearance and texture of the extrudate formulated with the incorporation of free VitD3 sample is likely to be caused by the presence of unencapsulated vitamin D3 in the product formulation.

Although its concentration is low at 0.5%, the hydrophobic free VitD3 solution could exclude from the extrudate matrix and move to the areas of higher shear in the extruder and the cooling die, acting as a lubricant at the metal surfaces in contact with the extrudate. This low-viscosity fluid lubricates those surfaces, causing some slippage that disrupts the continuous flow and affects the uniformity and consistency of the extruded product.

Furthermore, due to its hydrophobic nature, free VitD3 is likely to interfere with protein–protein interactions, weakening the texturization of the pea protein and preventing the formation of an elongated fiber network. As a result, breakable and non-texturized structure forms. These findings are consistent with the fiber texturization measurements presented in Table 1, which indicate that the formulation containing free VitD3 reduced fiber formation.

In contrast, using BSY-VitD3 mitigated these negative effects, demonstrating that vacuum impregnation protects the vitamin during processing and facilitates its incorporation in the formulation and processing. Encapsulating VitD3 in BSY helps maintain the structural integrity of the extruded product by preventing the free vitamin from interacting directly with the pea protein matrix, thereby preserving the formation of a protein fibrillar structure and enhancing the overall texture of the product.

These results align with previous findings, presenting similar surface characteristics in extruded yeast protein and pea protein formulations processed with high-moisture extrusion (HME), where the control and formulations containing yeast protein had comparable appearances [22]. In contrast, the extruded pea protein isolates exhibited cracked and flaky surfaces under similar extrusion conditions, whereas the addition of *Penicillium limosum* mycoprotein at a 5% concentration produced a smoother texture, without flaking or cracking, highlighting the potential of incorporating additional protein sources or encapsulated nutrients in a high-protein matrix like BSY to improve product quality [25]. In order to mimic the textural properties of different foods, a second protein such as zein can be incorporated into a pea protein matrix under alkaline conditions [26]. These results were applied for the HME of similar formulations [27].

In summary, incorporating BSY-VitD3 into plant-based meat analogs via extrusion provides a promising strategy for fortifying products with vitamin D while maintaining desirable textural properties. Vacuum impregnation effectively prevents the negative impact of free VitD3 on the extrusion process, supporting the formation of a stable and fibrous structure.

#### 3.1.1. Texture Profile

Texture encompasses a group of physical properties of a food product that determine how its structure deforms and breaks under external forces. Key parameters used to evaluate texture include hardness, cohesiveness, elasticity, gumminess, and chewiness. Additionally, the fibrous level of the product is an important functional attribute of proteins that influences both the processing behavior and the texture of meat analogs [25]. The texture profile analysis of the extruded products is summarized in Table 1.

Interestingly, no consistent trend was observed in texture attributes with the addition of BSY-VitD3 or free VitD3. The BSY-VitD3 sample showed reduced values across all parameters compared to the control, while the sample containing free VitD3 displayed increased resilience and springiness, with a reduction in hardness, gumminess, chewiness, and fibrous level.

Hardness, which is the peak force required to compress the product, and chewiness, which measures the effort needed to chew the product, are critical indicators of the product’s textural quality. Chewiness is a combined measure that reflects the relationship between gumminess and springiness [28,29].

Previous studies have shown similar textural effects when yeast was incorporated into protein matrices. In mixtures of yeast and pea protein (10–50%), hardness, chewiness, and fibrous level reductions were observed, while springiness remained unaffected [22]. However, adding 10% yeast protein in soy protein formulations increased hardness, reduced springiness, and did not significantly affect chewiness or the fibrous level [30]. Moreover, incorporating 10% starch fractions into pea protein extrudates produced similar effects as observed with the addition of BSY-VitD3, leading to reductions in all textural parameters [31].

Textural properties are generally positively correlated with protein content in both pea and soy extruded products [29]. The improvement in extrudate quality is often attributed to modifications in the disulfide cross-linking between protein molecules during extrusion. Disulfide bonds, which stabilize protein structures, are broken during extrusion, exposing active protein groups that form new non-covalent bonds as the product cools, enhancing the degree of cross-linking [32]. However, the cell walls of *Saccharomyces species*, particularly *S. pastorianus*, is composed predominantly of polysaccharides such as mannoproteins and glucans [11,33]. When raw materials contain high levels of polysaccharides, the interactions during extrusion may be more affected by protein–polysaccharide interactions rather than protein–protein interactions [29]. The addition of 2% BSY likely facilitated the formation of weaker intermolecular interactions, reducing all textural properties, including both lengthwise (parallel) and crosswise (perpendicular) structural strengths, as evidenced by changes in the fibrous grade.

Furthermore, the BSY used in this study was impregnated with vitamin D3. In other formulations, such as soy protein extrudates with added corn oil (a hydrophobic component), the hardness and chewiness of the extrudates decreased proportionally with increasing oil concentration [34]. This effect can be attributed to the presence of hydrophobic molecules within the extrudate matrix, which reduces the cross-linking density in the protein network, an effect noted by Isusi et al. (2023) [35]. This mechanism also may explain the more significant reduction in hardness and gumminess observed in the sample containing free VitD3, where the hydrophobic nature of the vitamin likely interfered with the protein’s structural organization.

Compared to the texture of cooked chicken breast, which has a reported hardness of 2405.2 ± 116.8 and chewiness of 1283.5 ± 34.0 [36], further optimization of the formulation—such as increasing the concentration of BSY-VitD3 and incorporating a fat source—could enhance the similarity of the plant-based product to animal-derived meat. Such modifications can potentially improve consumer preference and acceptance, bringing the texture of the extruded product closer to that of traditional meat products.

#### 3.1.2. Color

Color is an important attribute of extruded meat analogs, influencing consumer perception and acceptance. The color of these products is affected by the raw materials’ inherent hues and the chemical reactions occurring during extrusion, particularly the Maillard reaction [22]. The effects of BSY-VitD3 and free VitD3 on the color parameters (L*, a*, b*, and ΔEcontrol) in the extruded samples are summarized in Table 2, along with the CIE chroma (C*) and hue angle (h°) values. As anticipated, the addition of BSY-VitD3 (2%) had a more pronounced impact on all color parameters compared to the addition of free VitD3 (0.5%), which is likely due to the light-brown color of BSY, as shown in Figure S2 of the Supplementary Materials.

Previous studies using higher yeast biomass concentrations in pea- and soy protein-formulated extrudates have reported proportional increases in color intensity [22,30]. This suggests a direct relationship between yeast protein content and changes in color attributes. The presence of yeast biomass can promote reductions in lightness (L*), redness (a*), yellowness (b*), chroma (C*), and hue angle (h°), resulting in darker and more opaque samples, consistent with the visual appearance depicted in Figure 1. These effects can be attributed to the natural pigments present in yeast and their interactions with the protein matrix during the extrusion process, likely involving Maillard-type reactions that contribute to overall color development.

These findings enhance the novelty and practical relevance of this study for the development of functional plant-based foods, extending beyond the incorporation of free or yeast-encapsulated vitamin D. Overall, pea protein exhibits relatively stable color attributes across different formulations when compared to other legumes, such as lentils. For example, a recent study by Usman et al. (2023) [37] comparing germinated and non-germinated pea and lentil proteins found that lentil-based formulations resulted in brighter surfaces, as indicated by higher L* values. In our study, surface brightness was more pronounced in samples without yeast addition and was not significantly influenced by the presence of free vitamin D. This attribute is particularly important in meat analogues, as surface brightness contributes to the perception of freshness, thereby increasing consumer appeal. When compared to raw and cooked organic chicken thighs analyzed by Çapan and Bağdatli (2021) [38], where L* values decreased from 60.17 to 54.51 for brand D, and from 56.92 to 51.69 for brand F after cooking, the L* values observed in our extruded pea-based samples closely matched those of the cooked meat. This similarity supports the potential of these formulations for use in ready-to-eat plant-based products.

Furthermore, reductions in redness (a*) and yellowness (b*) were observed in yeast-containing samples. However, these values remained comparable to those reported for cooked chicken thighs (a* 6.12; b* 18.96 for brand D), further supporting their suitability for mimicking poultry products [38]. Textured legume-based proteins typically display a light-yellow hue due to the absence of strong natural pigments. The yellowish coloration observed in our samples enhances their ability to replicate the visual characteristics of poultry meat [37]. Nevertheless, higher yeast concentrations can compromise this resemblance by darkening and decreasing the brightness of the final product. For instance, extrudates formulated with 10% yeast in pea protein showed L 45.48 ± 1.00, a* 5.05 ± 0.44, and b* 15.98 ± 1.18 [39]. Therefore, optimizing the yeast concentration is crucial to achieve a balance between nutritional enrichment and desirable visual attributes, ultimately ensuring consumer acceptance of plant-based meat analogues.

#### 3.1.3. Water Activity and Moisture

**Figure 2:**
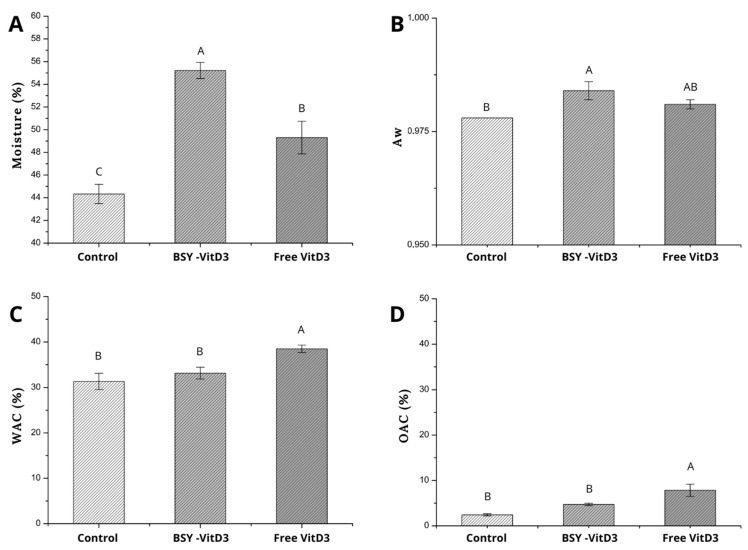
Mean and the standard deviation of (**A**) moisture content, (**B**) water activity, (**C**) water absorption capacity, and (**D**) oil absorption capacity of Plant-based extruded meat analog samples. Uppercase equal letters indicate no significant difference between the treatments (*p* > 0.05).

Moisture content is a key parameter in the extrusion process, determining the classification of final food products. Extrudates with moisture levels below 35% are defined as low-moisture products, while those with levels above 40% are considered high-moisture extrudates [22]. As shown in Figure 2A, all samples in this study are classified as high-moisture products, with the control sample exhibiting a moisture content of 44.33% ± 0.8, BSY-VitD3 at 55.22% ± 0.7, and free VitD3 at 49.30% ± 1.4. Furthermore, water activity (Aw) indicates the moisture status in extrudates, specifically reflecting the extent of water binding or the amount of free water present. The water activity of the samples investigated can also be considered high, as all values exceeded 0.90 (Control: 0.978 ± 0.000; BSY-VitD3: 0.984 ± 0.002; and free VitD3: 0.981 ± 0.001), as shown in Figure 2B. Higher water activity suggests a lower degree of water binding, enhancing the juiciness of the extrudate [40]. In addition, free water facilitates both chemical reactions and microbial growth that could affect the shelf life of these products.

Compared to the control, the extrudates’ moisture content and water activity containing BSY-VitD3 and free VitD3 increased (Figure 2A,B). The rise in moisture content in the sample containing yeast protein can be attributed to the formation of sheet structures and the exposure of tryptophan residues [22]. The increase in free water content due to the addition of yeast protein has been observed in extrudates formulated with soy protein by differential scanning calorimetry (DSC) [30]. This property is closely associated with the solubility characteristics of the raw material proteins and the chemical interaction of yeast proteins and vegetal proteins during the extrusion process [30,40].

The increase in moisture and water activity values observed with the addition of free vitamin D3 compared to the control may be attributed to the hydrophobic nature of that molecule, which affects water–protein and protein–protein interactions. A similar phenomenon was observed when maize oil as an O/W emulsion was used in formulations made from soy protein isolate (SPI) and wheat gluten (WG) processed by HME [41]. In these extrudates, the presence of oil in the formulations promoted an increased migration of both water and oil as the oil content increased, thereby reducing the water and oil binding properties.

#### 3.1.4. Water Absorption Capacity and Oil Absorption Capacity

As a complex food system, a key sought attribute of extruded meat analogs is their capacity to retain water and oil [25]. Water absorption capacity reflects how well the extrudate interacts with water. This capacity, which depends on the presence of hydrophilic groups and the number of polar sites, has a large influence on the extrudate mechanical properties. Conversely, oil absorption capacity is primarily determined by the concentration of hydrophobic groups on the extrudate’s surface, indicating how effectively the extrudate can absorb oil [42]. The water absorption capacity (WAC) and oil absorption capacity (OAC) of the Control (31.34 ± 1.79; 2.42 ± 0.25), BSY-VitD3 (33.16 ± 1.28; 4.73 ± 0.27), and Free VitD3 (38.49 ± 0.83; 7.84 ± 1.35) samples are illustrated in Figure 2C and 2D, respectively. A significant increase in both WAC and OAC was observed only in the sample supplemented with free vitamin D3.

Previously reported PPI-based extrudates with enhanced OAC exhibited an increased number of available hydrophobic sites following thermal extrusion while maintaining the number of hydrophilic sites [25]. The addition of yeast protein did not appear to affect the exposure of pea protein’s hydrophilic and hydrophobic sites. In contrast, in the current work, formulations containing free vitamin D3 resulted in extrudates with greater water and oil absorption. Cholecalciferol is a bioactive with moderate hydrophobicity, as well as curcumin and astaxanthin [42]. Studies on cholecalciferol interaction with the soluble glutelin pea protein fraction demonstrated that there is a lower rate dissociation of the protein–bioactive compound complexes at lower concentrations of the bioactive compound. Additionally, bioactive compounds with moderate lipophilicity (curcumin, astaxanthin, and cholecalciferol) exhibited stronger binding affinity [42]. The increased water and oil absorption observed in the extrudates containing free vitamin D3 may be related to the molecule’s chemical properties and its interaction mode with the protein, serving as a bridge for interactions with both water and oil.

### 3.2. Vitamin Retention

The high temperatures and intense shear forces involved in the extrusion process can significantly impact vitamin retention. Previous research has shown that the extent of vitamin loss was influenced by factors such as screw speed, r die diameter, and feed rates [43]. Table 3 presents the retention data of vitamin D3 immediately following the extrusion process and after 30 days of storage of the products at room temperature, comparing samples containing BSY-VitD3 with those containing free VitD3.

Over 50% of free vitamin D3 was lost during extrusion, indicating that the processing conditions are sufficiently harsh to significantly reduce its retention. However, for formulations in which vitamin D3 was impregnated into *S. pastorianus* (BSY), more than 90% of the vitamin was recovered post-processing, highlighting the yeast biomass’ protective potential for this bioactive compound. During storage, the variation in the percentage of vitamin D3 retention does not differ between samples, both being 6.5%.

Vitamins A, D, E, K, and C and folic acid are notably vulnerable to degradation during the extrusion process, with vitamin D showing a particular sensitivity to elevated temperatures [43,44]. Morin et al. (2021) [43] reported a retention rate of approximately 62 ± 14% for vitamin D in extrudates. Furthermore, the authors cite that fortified feeds for swine prepared using a twin-screw extruder (Brabender DSE-25) significantly increased vitamin D degradation as the extrusion temperature rose from 100 to 180 °C [43,44]. Vitamin D was found to be highly prone to oxidation, resulting in poor retention not only during extrusion but also throughout storage in extruded food products [3].

The effective microencapsulation of Vitamin D within pharmaceutical applications has opened avenues for its incorporation into food matrices, addressing the inherent low water solubility and chemical instability challenges of Vitamin D in such environments. The encapsulation techniques not only shield Vitamin D from physical and chemical degradation factors such as moisture, oxidation, pH fluctuations, temperature changes, and mechanical stress but also improve its bioavailability through controlled and targeted release mechanisms [3].

In the context of extruded products, the primary objective of microencapsulation is to protect the biomolecule during processing. The favorable outcomes observed with BSY-VitD3 are corroborated by other studies, which underscore the potential of encapsulation techniques. For example, research on extruded pig feed supplemented with VitD3 demonstrated that microencapsulation in hydrogenated fat using spray-drying led to a 21% increase in vitamin retention [44].

Although various methodologies exist for incorporating and protecting bioactive compounds, using residual yeast biomass offers a sustainable approach by promoting the circular use of byproducts from the fermentation industry [3,8,9,13]. Additionally, the structural characteristics of the yeast cell wall, which is rich in polysaccharide, may enhance the interaction and protection of hydrophobic molecules. According to FTIR data [9], the OH peak shifted to higher wavenumbers in cholecalciferol-loaded yeast microcapsules. This shift may be attributed to increased hydrogen bonding between yeast cells and cholecalciferol. Furthermore, Costa et al. (2024) [10] indicated that FTIR provides a good indication of the yeast cell wall composition, suggesting that vitamin D3 showed greater affinity for intact yeast cell walls, particularly observed in the absorption bands related to the lipid composition of yeast. This data indicated that, given its non-polar nature, vitamins interacted with the lipids of the yeast cell membrane. Hydrogen bonding and hydrophobic interactions likely play a significant role in incorporating cholecalciferol into the yeast cell matrix and in its protection against stress and temperature factors, as observed in this study.

## 4. Conclusions

This study demonstrates that vacuum impregnation of BSY with vitamin D3 effectively facilitates the incorporation of this nutrient into a plant-based meat formulation. It protects vitamin D3 throughout the extrusion process, providing a simple, fast, and cost-effective method for creating fortified extruded products.

Adding 2% of BSY-VitD3 (*w*/*w*) reduces all texture profile parameters, including hardness, resilience, cohesion, springiness, gumminess, chewiness, and fibrousness. In contrast, a formulation containing 0.5% free vitamin D3 (*w*/*w*) enhances the extruded product’s capacity to absorb water and oil.

These results highlight the BSY ability to protect bioactive compounds in a complex food system and open new possibilities for developing plant-based meat analogs that incorporate sensitive bioactive molecules and other ingredients prone to deterioration during extrusion.

## Figures and Tables

**Table 1:** Textural properties of extruded plant-based meat products.

Texture Profile Analysis	Extruded Plant-Based Meat Products		
	CONTROL	BSY-VitD3	FREE VitD3
Hardness (N)	14,231.5 ± 672.8 ^a^	8883.5 ± 54.6 ^b^	4163.6 ± 305.0 ^c^
Resilience (%)	6.1 ± 0.08 ^b^	3.5 ± 0.11 ^c^	7.5 ± 0.4 ^a^
Cohesion (N)	0.2 ± 0.00 ^a^	0.14 ± 0.00 ^b^	0.2 ± 0.02 ^a^
Springiness (%)	14.8 ± 0.03 ^b^	12.8 ± 0.11 ^c^	18.2 ± 0.72 ^a^
Gumminess	2775.9 ± 145.97 ^a^	1260.7 ± 38.64 ^b^	951.3 ± 64.60 ^c^
Chewiness (N)	438.0 ± 12.29 ^a^	163.7 ± 7.07 ^b^	186.6 ± 2.09 ^b^
Fibrous degree	2.0 ± 0.07 ^a^	1.4 ± 0.04 ^b^	1.1 ± 0.17 ^b^

Lowercase identical letters in the same line indicate no significant difference between the treatments (*p* > 0.05).

**Table 2:** Color parameters of extruded samples.

Colors Parameters	Samples		
	CONTROL	BSY-VitD3	FREE VitD3
L*	53.3 ± 0.75 ^a^	50.3 ± 0.16 ^b^	51.4 ± 0.97 ^ab^
a*	10.4 ± 0.31 ^a^	6.3 ± 0.16 ^c^	7.5 ± 0.10 ^b^
b*	25.0 ± 0.95 ^a^	20.0 ± 0.08 ^c^	23.2 ± 0.49 ^b^
C*	26.1 ± 1.04 ^a^	21.04 ± 0.09 ^c^	24.9 ± 0.50 ^b^
h°	68.13 ± 0.49 ^b^	72.28 ± 0.32 ^a^	72.12 ± 0.60 ^a^
Visual nuances	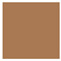	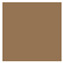	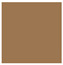
ΔE_control_	—	7.2 ± 0.53 ^a^	4.2 ± 1.20 ^b^

Lowercase equal letters in the same line indicate no significant difference between the treatments (*p* > 0.05).

**Table 3:** Vitamin retention (%) after extrusion and product storage.

Vitamin D3 Retention (%)	Samples	
	BSY-VitD3	FREE VitD3
**Immediately after the extrusion process**	91.6 ± 1.48 ^A,a^	45.4 ± 1.14 ^B,a^
**After 30 days of storage**	85.1 ± 1.48 ^A,b^	38.9 ± 1.21 ^B,b^

Data are expressed as mean ± standard deviation. Equal uppercase superscript letters indicate no significant difference between treatments (BSY VitD3 and FREE VitD), and equal lowercase superscript letters indicate no significant difference between analysis times (immediately after the extrusion process and after 30 days of storage) (*p* > 0.05).

## Data Availability

The original contributions presented in the study are included in the article/Supplementary Material, further inquiries can be directed to the corresponding author.

## Supplementary Materials

The following supporting information can be downloaded at: https://www.mdpi.com/article/10.3390/foods14091500/s1, Figure S1—Standard curve for vitamin D3. The graph was plotted considering the independent duplicate. The line was projected by considering the deviations between the data points and the intersection of the axes at the origin (0,0). The line with Pearson’s R of 0.99962 and R2 of 0.992 was accepted. The slope was 157852.72 with a standard deviation of 1082.12, and the intercept was −95546.47 with a standard deviation of 40858.14; Figure S2—BSY-VitD3. The powder of S. pastorianus vacuum-impregnated with Vitamin D.
