# Supplementary material for: Production of Vitamin D3-Fortified Plant-Based Meat Analogs Through High-Moisture Extrusion
Source: Foods. 2025 Apr 25;14(9):1500. doi: 10.3390/foods14091500 (PMC12071410; doi:10.3390/foods14091500)
Supplement: Supplementary file 1 [file foods-14-01500-s001.zip › foods-3597203-supplementary.pdf]

Supplementary material

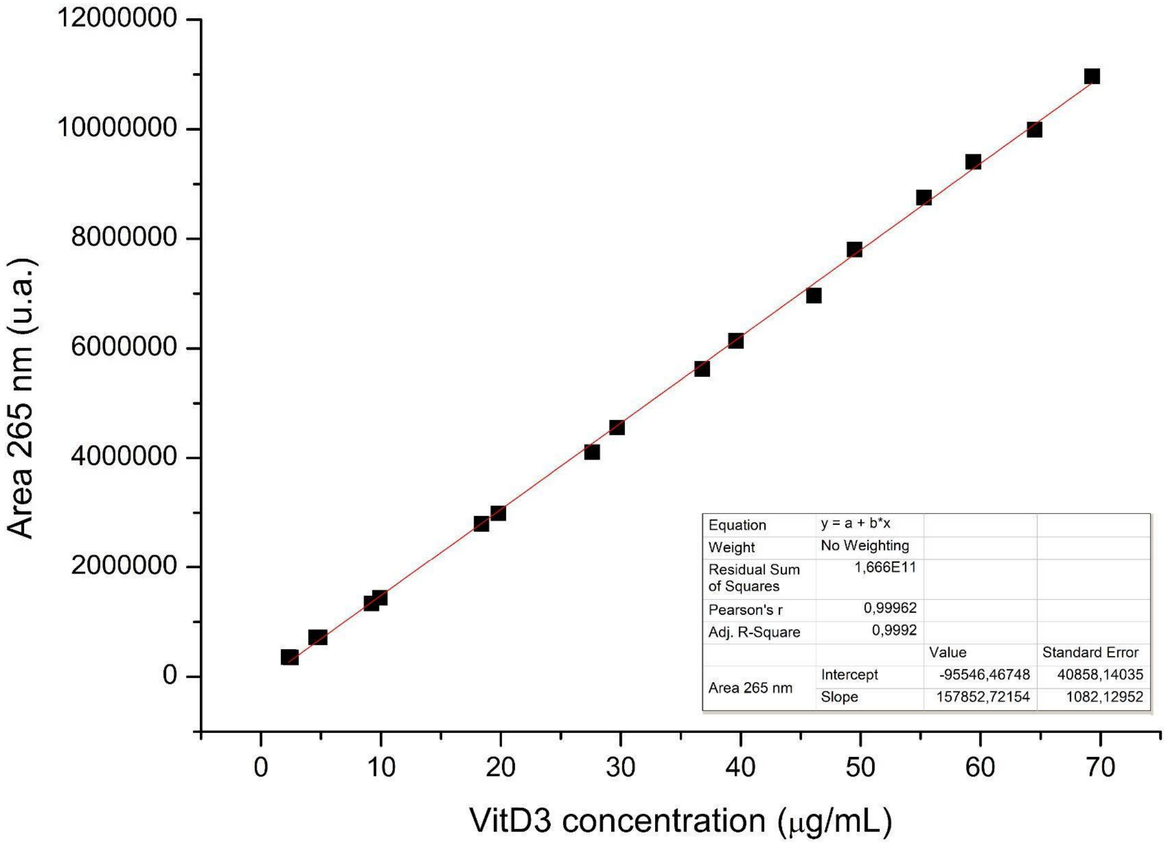

	A	B	C	D
1	Equation	y = a + b*x		
2	Weight	No Weighting		
3	Residual Sum of Squares	1,666E11		
4	Pearson's r	0,99962		
5	Adj. R-Square	0,9992		
6			Value	Standard Error
7	Area 265 nm	Intercept	-95546,46748	40858,14035
8		Slope	157852,72154	1082,12952

Figure S1 - Standard curve for vitamin D3. The graph was plotted considering the independent duplicate. The line was projected by considering the deviations between the data points and the intersection of the axes at the origin (0,0). The line with Pearson's r of 0.99962 and R^2 of 0.992 was accepted. The slope was 157852,72 with a standard deviation of 1082,12 and the intercept was -95546,47 with a standard deviation of 40858,14.

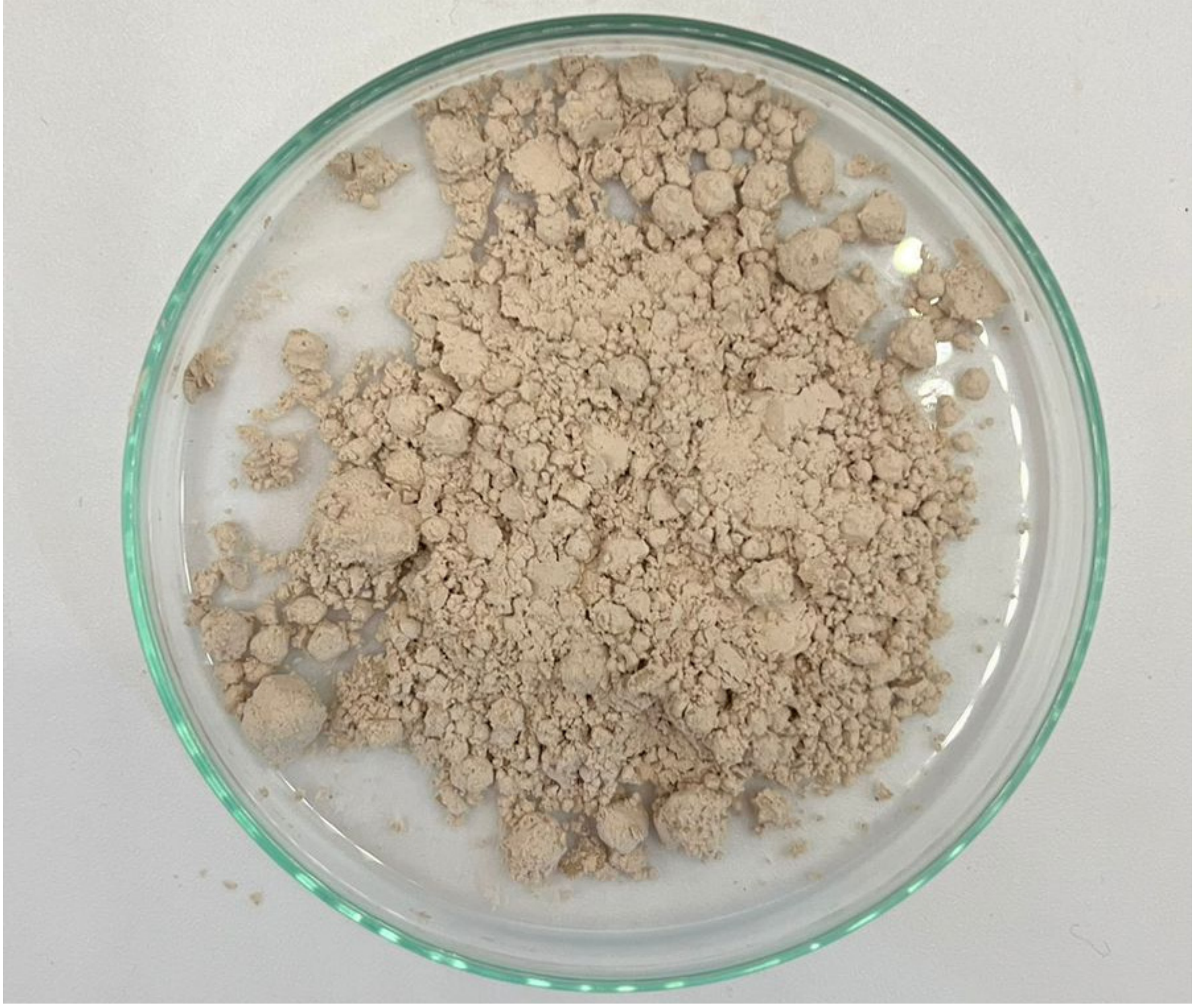

Figure S2. BSY-VitD3 or powdered biomass of *S. pastorianus* vacuum-impregnated with Vitamin D3.
